# Integrative Analysis of Genetic Network and Brain Imaging using Generative Models for Alzheimer’s Disease Prediction

**DOI:** 10.1002/alz.091830

**Published:** 2025-01-09

**Authors:** Dong‐gi Lee, Sang‐Hyuk Jung, Li Shen, Dokyoon Kim

**Affiliations:** ^1^ Perelman School of Medicine, University of Pennsylvania, Philadelphia, PA USA; ^2^ Institute for Biomedical Informatics, University of Pennsylvania, Philadelphia, PA USA

## Abstract

**Background:**

The structural characteristics of the brain, specifically the decrease of individual gray (or white) matter volumes, provide valuable insights into brain function and cognitive decline, including the development of Alzheimer’s disease (AD). In addition, genetic factors can play a significant role in changes in brain volumes, influencing biological activities and interacting in complex ways. In this study, we aim to investigate the relationship between genetic factors, structural brain volume, and the risk of AD.

**Method:**

The proposed method utilizes a SNP network to capture interactions between genetic variants based on linkage disequilibrium scores. The network represents the complex relationships among genetic factors. Feature propagation, a technique that refines genetic variant information based on graph neural networks (GNNs), is then employed to predict AD risk scores. Meanwhile, brain imaging endophenotypes, following the concept of transcriptome‐wide association studies (TWAS), are used to enhance prediction power and identify significant regions affecting specific diseases. Moreover, a generative artificial intelligence model is applied to produce pseudo‐imaging endophenotypes for samples with only genetic information.

**Result:**

The proposed method was applied to data including (i) both genetic and brain imaging information from two datasets: UK Biobank (UKBB), Alzheimer's Disease Neuroimaging Initiative (ADNI), and (ii) only genetic information from the All of Us research program (AoU). The results were cross‐validated from the two datasets (UKBB and ADNI), and a generative model was applied to AoU. The final prediction results were compared with other models, including the polygenic risk score–continuous shrinkage (PRS‐CS) model. Compared to the other methods, the proposed method demonstrated superior prediction performance and revealed genetic variants and brain regions that impact AD.

**Conclusion:**

By integrating genetic information and brain imaging data, this study achieved improved disease risk prediction and a deeper understanding of the phased mechanism of AD associated with genetic variants and brain lesions. Furthermore, the insights gained into the relationship between genetics, brain structure, and diseases are expected to contribute to advancing our knowledge of disease progression and inform future research and clinical strategies for the prevention and management of these conditions.

